# Detection of Microbial Contamination in Nanomaterials Using LAL, rFC and Cell-Based Assays: Implications for Nanotoxicological Hazard Assessment

**DOI:** 10.3390/nano15241871

**Published:** 2025-12-13

**Authors:** Peng Lei, Fikirte Debebe Zegeye, Mayes Alswady-Hoff, Chiara Marcolungo, Pernille Høgh Danielsen, Anne Mette Madsen, Håkan Wallin, Ulla Vogel, Shan Zienolddiny-Narui, Johanna Samulin Erdem

**Affiliations:** 1 National Institute of Occupational Health, NO-0363 Oslo, Norway; 2 Department of Industrial Engineering, University of Padova, 35122 Padova, Italy; 3 National Research Centre for the Working Environment, DK-2100 Copenhagen, Denmark

**Keywords:** nanotoxicology, nano safety, bacterial contamination, nanoparticles, inflammation, endotoxin, toll-like receptor

## Abstract

Accurate detection of microbial contamination is essential in the assessment of toxicological and immunological responses to various materials, as low-level contaminants can lead to confounding results. Traditional endotoxin testing relies on the Limulus Amebocyte Lysate (LAL) assay, which depends on horseshoe crab blood and raises both ecological and ethical concerns. Sustainable alternatives such as recombinant Factor C (rFC) provide a promising solution, yet validation for the detection of endotoxin in nanomaterials remains incomplete. In this study, we have used rFC alongside Toll-like receptor (TLR) reporter assays to detect both endotoxin and broader microbial contaminants in 31 nanomaterials from diverse classes. Special attention was given to assay interference by nanomaterials to ensure reliable detection. The rFC assay demonstrated a sensitive detection limit of 0.005 EU/mL, equivalent to the LAL assay, and showed that more than 50% of tested nanomaterials contained low-level endotoxin contamination. Additionally, several nanomaterials activated the TLR2 reporter, indicative of microbial contaminants beyond endotoxin. These results suggest that rFC can serve as a sustainable and reliable replacement for LAL in nanomaterial endotoxin testing but also emphasize the limitations of relying solely on endotoxin-specific assays. We recommend that future nanotoxicological evaluations integrate rFC with complementary methods, such as TLR-based approaches, and include thorough interference controls to ensure robust and comprehensive microbial contamination assessment.

## 1. Introduction

The impact of engineered nanomaterials on biological processes and, consequently, their implications for human health and the environment are important to the nanotechnology industry, regulators, and scientists. Occupational exposure to engineered nanomaterials has been associated with adverse health effects, particularly in the respiratory system. Pulmonary inflammation and fibrosis are among the most consistently reported outcomes in both experimental models and exposed workers, with oxidative stress and inflammation recognized as key mechanistic pathways [1,2,3,4,5]. These effects can arise not only from the intrinsic properties of the nanomaterials themselves but also from extrinsic factors, e.g., microbial contamination. Assessment of contamination is critical as even minute amounts of microbial contamination may influence the toxicological responses of nanomaterials, resulting in an over- or underestimation of toxic effects [6]. In occupational hazard assessment, unrecognized contamination can lead to inappropriate hazard classification and risk management decisions. Therefore, risk assessment of engineered nanomaterials produced and handled in occupational environments requires correct evaluation of potential contamination and inclusion of optical-interference controls in alignment with internationally recognized quality-control standards within pharmaceutical manufacturing, food safety, and occupational air monitoring [7,8,9].

Microbiological contamination of nanomaterials is common and may occur during production, prolonged storage, or handling [10]. Microbial components of Gram-negative bacteria are well-recognized contaminants of nanomaterials acknowledged to influence inflammatory and immune responses. Surprisingly, reports have shown that between 30 and 40% of nanomaterials associated with EU-funded nanomedicine-related projects have endotoxin levels above the regulatory requirements for preclinical assessment [11]. Endotoxin is heat stable and cannot be easily removed by common sterilization techniques such as autoclaving and ionization radiation [12]. Keeping this in mind, several recent reviews have stressed the importance of sufficient endotoxin testing to avoid misinterpretation of toxicological endpoints [11,13]. Despite this, reporting of endotoxin contamination is often lacking, severely diminishing the potential relevance of nanotoxicological findings. This is exemplified by findings in a recent systematic review on TiO_2_ nanoparticles evaluating effects such as inflammation, oxidative stress, and genotoxicity, showing that less than 30% of the included studies had considered endotoxin contamination [14].

The assessment of microbial contaminations in nanomaterials remains technically challenging. More robust testing strategies need to be developed, and limitations of existing strategies should be carefully considered when interpreting the results. For endotoxin testing, the chromogenic, turbidimetric, or gel clot Limulus amoebocyte lysate (LAL) assays are most used due to their relatively fast, sensitive, and specific detection. However, due to ethical and sustainability concerns, the U.S. Food and Drug Administration in 2012 [15] published a new guidance recommendation suggesting two new methods, namely the monocyte activation test (MAT) [16] and the recombinant Factor C (rFC) assay [17,18] as alternative methods for endotoxin and pyrogen testing [11]. Notably, the MAT assay measures inflammatory effects by assessing pro-inflammatory cytokine secretion and is thus not specific to endotoxin but detects any inflammatory responses, including potential nanomaterial-specific effects [12]. On the contrary, rFC utilizes a synthetic enzyme for endotoxin detection and can be considered a direct substitute for the LAL assay, making it a more suitable alternative for endotoxin testing of nanomaterials. In addition to these regulatory accepted methods for endotoxin testing, several alternative approaches have been suggested, including the Toll-like receptor 4 (TLR4) reporter assay, high performance liquid chromatography and mass spectrometry (HPLC-MS) methods, and macrophage activation test [11]. Each of these approaches have strengths and limitations when applied to nanomaterials. TLR4 reporter assays offer high biological relevance but rely on living cells and can be affected by cytotoxicity. Furthermore, some evidence suggests that nanomaterials may directly interact with cell surface receptors and elicit inflammatory signaling [19]. HPLC–MS provides structural information yet is limited by extraction efficiency and matrix effects, while the macrophage activation test, similar to MAT, is easily confounded by nanomaterial-induced inflammation. Moreover, biochemical tests, such as LAL and rFC methods, quantify only dispersed endotoxins, while cell-based detection methods sense biologically available, including particle-associated, contaminants, and may provide complementary information to the classical LAL or rFC assays. These methods may prove to be valuable substitutes in the future but need validation and thorough evaluation of aspects such as interference, sensitivity, and specificity.

While there is now a general acceptance of the need for endotoxin contamination assessment in nanotoxicological studies, the potential effects of other microbial contaminants have, at large, been underestimated or ignored in the assessment of nanomaterial toxicity. To date, no assessment methods have been thoroughly investigated or validated for the detection of other microbial contaminants, such as Gram-positive contaminants or fungi, on nanomaterials. Furthermore, nanomaterials may interfere with fluorometric and absorbance-based measurements or directly interact with proteins or reagent components, thereby affecting assay readouts, resulting in insufficient, inconclusive, or misleading results. In addition, large interlaboratory variations in endotoxin measurements have been reported [20], and nanomaterials often interfere with traditional endotoxin tests [21]. Altogether, highlighting a need for multiple assessment methods and established standardized protocols for interference control. Thus, improved methods to detect microbial contamination are urgently needed to ensure that observed responses genuinely reflect nanomaterial-related toxicity rather than artifacts of experimental impurity.

This study aimed to systematically evaluate microbial contaminations across 31 different nanomaterials representing the major classical nanomaterial classes, i.e., metal and metal oxides, carbon-based materials, including graphene, carbon black and carbon nanotubes, and nanoclays. The suitability of the rFC and TLR reporter assays as detection methods of microbial contamination was assessed, and nanomaterial interferences were thoroughly investigated. Our data suggest that TLR reporter assays hold promise as complementary tools for assessing microbial contamination of nanomaterials. These findings highlight the urgent need for standardized contamination assessment approaches that employ multiple testing methods and include appropriate interference controls, ensuring accurate interpretation of nanomaterial-induced immunological responses.

## 2. Materials and Methods

### 2.1. Materials and Characterization

Material specifics and characterization data are summarized in Table 1. The NANOGENOTOX dispersion protocol, with modifications, was applied for particle dispersion for reporter cell exposure [22]. Briefly, 1.28 mg/mL stock solutions were prepared by dispersing 7.68 mg of nanomaterials in 6 mL of 0.05% (*v*/*v*) BSA/endotoxin-free water (Merck, St. Louis, MO, USA, and Thermo Fisher Scientific, Waltham, MA, USA). The dispersion was sonicated on ice for 16 min at 10% amplitude using a Branson Sonifier S-450D (Branson Ultrasonics Corp., Danbury, CT, USA), immediately prior to exposure. The hydrodynamic diameter of the particles in dispersion media (DLS stock) and exposure media at the time of exposure (DLS media) and after 24 h (DLS media 24 h) was assessed by dynamic light scattering (DLS) (Zetasizer Nano, Malvern Instruments, Ltd.; Worcestershire, UK) (Table 1). Comprehensive physicochemical datasets for these same materials have been published previously, including CNT surface functionalization/metal content and nanoclay surface modifier characterization [2,23,24,25,26,27,28,29,30,31,32,33,34,35].

### 2.2. Measurement of Endotoxin by Recombinant Factor C

The endotoxin levels in all nanomaterials were assessed using PyroGene^®^ recombinant Factor C (rFC) in vitro assay (Endozyme^®^ II GO, bioMérieux, Marcy-l’Étoile, France). The assay uses a pre-filled GOPLATE™ containing the standard curve and positive product controls, ensuring consistent preparation across plates and reducing handling errors. According to the manufacturer, the assay provides lot-to-lot reproducibility, high endotoxin specificity (no Factor G pathway), and a substantially reduced invalid rate compared with classical LAL formats. Plate validity required a standard curve with R^2^ ≥ 0.99, spike-recovery between 50 and 200%, and an acceptable negative-control background. For rFC analysis, 3 to 4 mg of nanomaterial was dispersed in 6 mL endotoxin-free water using Bandelin Sonorex RK510H ultrasonic bath (BANDELIN electronic GmbH & Co. KG, Berlin, Germany) for 20 min. This step effectively releases surface-associated endotoxin into the aqueous phase before rFC detection. Extracted endotoxin was assessed in nanomaterial dispersions at the final concentrations of approximately 100 µg/mL or 10 µg/mL (only for NM110 and Bentonite). The standard curve was made using a serially diluted lipopolysaccharide (LPS, Lonza, Basel, Switzerland), and the predicted endotoxin concentration of the tested materials was calculated based on the standard curve equation and plotted on the standard curve. All results were normalized with respect to the nanoparticles’ weight. Following the current international standards for endotoxin testing, the rFC assay included inhibition-enhancement controls (IECs), which are spike-recovery controls consisting of a known concentration of endotoxin (0.5 EU/mL) spiked into the test samples to determine their ability to inhibit or enhance the assay readout. An inhibition is considered a spike-recovery < 50% of the concentration introduced, whereas an enhancement is defined as > 200%. In addition, interference was assessed with spiked-in nanomaterials in the absence of rFC enzyme to assess the potential interference with absorbance readouts. The spiked-in nanomaterial concentrations represent a worst-case scenario, assuming complete (100%) deposition and cellular uptake of the administered nanomaterial dose. Each spiked and unspiked sample was measured in duplicates.

### 2.3. Assessment of Microbial Contamination by Reporter Cell Assays

To assess microbial contamination, the HEK-Blue™-hTLR2 and HEK-Blue™-hTLR4 reporter cell lines, together with their parental control HEK-Blue™ Null cell line (InvivoGen, Toulouse, France), were utilized. The cells are derived from human embryonic kidney (HEK293) cells and contain an NF-κB-inducible secreted embryonic alkaline phosphatase (SEAP) gene reporter construct. The HEK-Blue™-hTLR2, HEK-Blue™-hTLR4 cells overexpress TLR2 and TLR4, respectively. The cells were cultured in high-glucose DMEM (Gibco, Thermo Fisher Scientific; Waltham, MA, USA) supplemented with 10% Ultra-Low Endotoxin Fetal Bovine Serum (Biowest, Bradenton, FL, USA), containing 100 units/mL penicillin and 100 µg/mL streptomycin (Gibco), and Normocin (InvivoGen). Zeocin (InvivoGen) was added to the culture medium for HEK-Blue™ Null1 cells, while HEK-Blue™ selection antibiotics (InvivoGen) were used for HEK-Blue™-hTLR2 and HEK-Blue™-hTLR4 cells for plasmid maintenance purposes. All cells were maintained at 37 °C in a humidified incubator with 5% CO_2_, and experiments were performed using cells within passage 20, in accordance with InvivoGen’s recommendations.

Assessment of nanomaterial contamination was performed in 96-well plate format (Sarstedt; Nümbrecht, Germany; growth area: 0.29 cm^2^). Cells were seeded in triplicate at a density of 280,000 cells per well in a final volume of 180 µL and incubated for 24 h. Cells were then exposed to 20 µL dispersed nanomaterials at the final concentrations of 10 µg/mL or 100 µg/mL for 24 h. For NM110 and Bentonite, only the lower concentration was used as previous data showed reduced cell viability at high concentrations [3,36]. Moreover, NRCWE-022 was assessed only at the lower concentration, based on prior internal data from the SmartNanoTox consortium (confidential, unpublished). Media and cell cultures were incubated with nanomaterials at 37 °C for 24 h and examined by light microscopy to confirm the absence of viable bacterial contamination and growth. Standard curves using positive controls included purified lipoteichoic acid (LTA) from *Staphylococcus aureus* (InvivoGen, tlrl-pslta) and ultrapure LPS from *Escherichia coli* strain O55:B5 (InvivoGen, tlrl-b5lps) to activate TLR2 and TLR4, respectively. The standard curve was made using respective positive controls, and the predicted contaminant concentration of the tested materials was calculated based on the standard curve equation and plotted on the standard curve. Endotoxin-free water was used as a negative control. Interference of the nanomaterials with the HEK-Blue™ TLR reporter cell assay was assessed using spiked controls as described by Bohmer and colleagues [37]. In short, untreated cells and positive control cells were treated with either media or LTA/LPS. 20 µL of the cell culture supernatant was carefully transferred to a new 96-well plate, and nanomaterials were added to the collected cell culture supernatants at a worst-case scenario of 100 µg/mL. For materials showing interference at the worst-case scenario, the nanomaterials were spiked in using a titration series of 100 µg/mL, 50 µg/mL, and 10 µg/mL to establish a lower, non-interfering threshold for accurate endotoxin assessment. Following the addition of nanomaterials, the media was mixed with 180 µL of QUANTI-Blue™ Solution. The plate was incubated at 37 °C for 3 h, and the SEAP levels were determined by measuring the absorbance at 649 nm using a Synergy Neo2 multi-mode reader (BioTek Instruments, Winooski, VT, USA). Interference was defined as a ±20% change in the readout signal compared to the control. The results were normalized to the weight of the analyzed nanoparticles. Each sample was measured in duplicates. The nanomaterial-free dispersion buffer (0.05% BSA in endotoxin-free water) was included as the negative control in all QUANTI-Blue measurements, ensuring that any background interaction between the buffer and the detection reagent was fully accounted for.

### 2.4. Data Analysis and Statistical Analysis

The limit of detection (LOD) for TLR reporter assays was determined as two times the standard deviation. For the rFC assay, the LOD was determined at 0.05 EU/mL (equivalent to 0.005 ng/mL), based on the calibration data showing that a 0.005 EU/mL standard yielded the same readouts as background (unexposed controls). Measurements above the LOD were interpreted as positive. Statistical analyses were conducted using one-way ANOVA followed by Šídák’s multiple comparisons test (α = 0.05) to correct for multiple testing. Analyses were performed in GraphPad Prism (version 10.5.0.0; GraphPad Software, San Diego, CA, USA). Adjusted *p*-values < 0.05 were considered statistically significant. Correlation between endotoxin levels and TLR activation was assessed using Pearson correlation.

## 3. Results

### 3.1. Recombinant Factor C Assay

**Figure 1:**
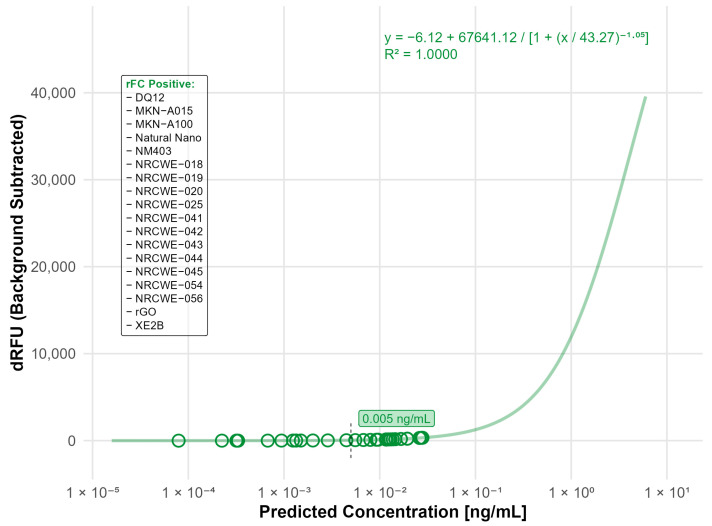
Nanomaterials identified as endotoxin positive in the rFC analysis. The standard curve was fitted using a four-parameter logistic (4PL) model (R^2^ = 0.9994) with a limit of detection (LOD) of 0.005 ng/mL. The limit of detection (LOD) is indicated by the black dashed line. Open circles indicate predicted endotoxin concentrations of tested nanomaterials. Materials listed in the frame were identified as endotoxin-positive based on responses above the LOD.

Assessment of interference by inhibition-enhancement controls (IECs) included in the commercial assay and by additional assessment of spectral interference in the absence of enzyme showed that none of the nanomaterials interfered with the rFC assay (Table S1). Endotoxin measurement resulted in detectable endotoxin levels (LOD: 0.005 ng/mL, equal to 0.05 EU/mL) in 18 of the tested materials, more than half of the total materials tested. While the levels of endotoxin were generally low, the highest contamination values were measured for rGO (0.028 ng/mL), the single-walled carbon nanotubes (SWCNTs) (NRCWE-054: 0.027 ng/mL and NRCWE-056: 0.019 ng/mL), the SiO_2_ nanomaterial (DQ12: 0.026 ng/mL), and the Fe_2_O_3_ nanomaterials (NRCWE-018: 0.0194 ng/mL and NRCWE-019: 0.0145 ng/mL) (Figure 1).

### 3.2. Particle Characterization by DLS

To investigate the stability of the nanomaterials used in cell-based assays, DLS was assessed in the dispersion and cell culture media at 0 and 24 h (Table 1). Most nanomaterials showed relatively high dispersion stability with minor changes in Z-average values in the media over the 24 h incubation time, with PDI values around 0.4, indicating moderate aggregation. In contrast, several high-aspect-ratio CNTs (including Mitsui-7, NM401, NM403, NRCWE-040, -043, -044, and -045) had reduced Z-averages and PDIs after 24 h. The apparent decrease in Z-average and PDI after 24 h is likely an artifact of sedimentation of larger CNT agglomerates out of the DLS detection limit rather than true re-dispersion of the materials.

### 3.3. TLR4 Reporter Assay

**Figure 2:**
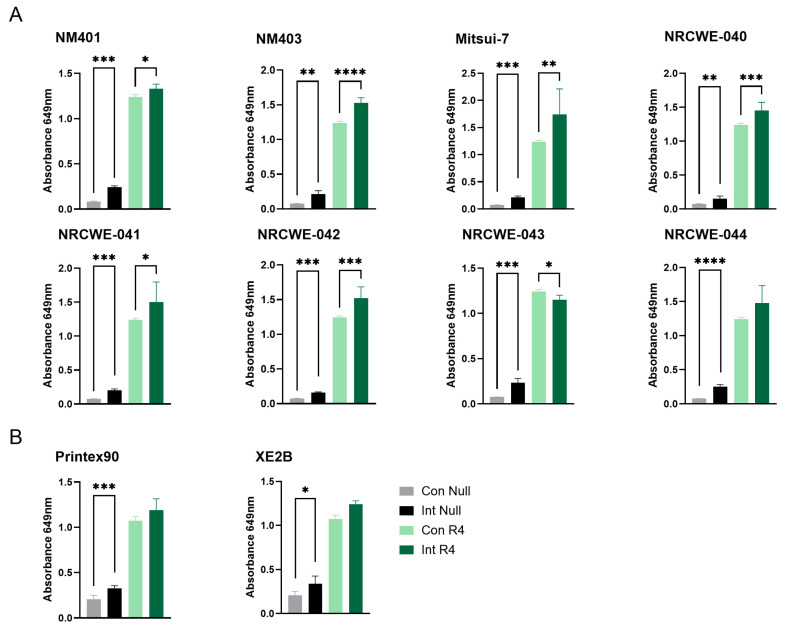
Interference of carbon-based nanomaterials with the TLR4 reporter assay. Interference was assessed for (**A**) MWCNTs and (**B**) carbon black materials at a concentration of 100 µg/mL. Con Null and Int Null represent negative control cells without a receptor agonist, in the absence or presence of nanomaterials, respectively. Con R4 and Int R4 represent TLR4 reporter cells stimulated with agonist, without or with nanomaterials, respectively. Data are presented as mean ± SD (*n* = 3). Only statistically significant changes that also exceeded a 20% difference relative to the respective control are shown in the figure. Statistical significance compared with respective controls is indicated (* *p* < 0.05, ** *p* < 0.01, *** *p* < 0.001, **** *p* < 0.0001). Con = Control, Int = Interference.

For the TLR4 reporter assay, significant interference was observed for all the included multi-walled carbon nanotubes (MWCNTs) as well as the carbon black materials, namely NM401, NM403, Mitsui-7, NRCWE-040, NRCWE-041, NRCWE-042, NRCWE-043, NRCWE-044, NRCWE-045, Printex90, and XE2B (Figure 2A,B). Both material types significantly increased the baseline absorbance in the absence of the receptor agonist, and most of them further enhanced the assay response upon agonist stimulation. The interference was consistent even at diluted nanomaterial concentrations (MWCNTs, Figure S1), though the higher concentration of materials exhibited stronger interference, suggesting that intrinsic optical or chemical properties of these carbon-based materials interfere with the assay measurement, potentially confounding endotoxin quantification. No interference or limited interference was observed for the remaining nanomaterial classes (Figure S2), suggesting that the TLR4 reporter assay is suitable for assessment of microbial contamination for SWCNT, metal-oxide, nanoclay, and graphene oxide materials.

**Figure 3:**
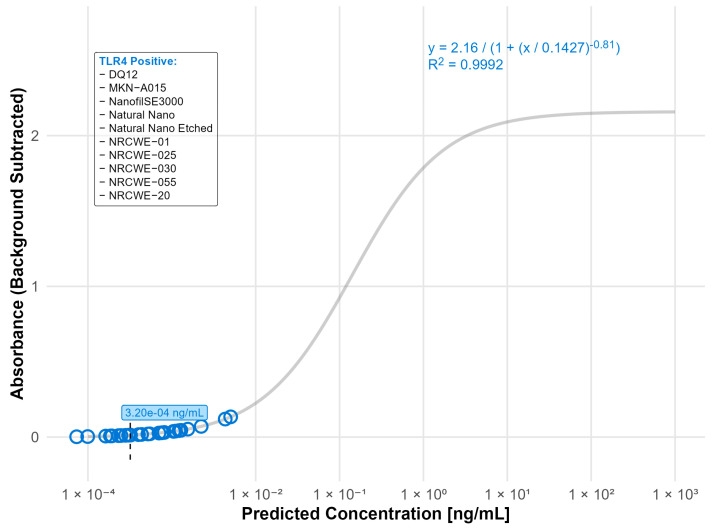
Nanomaterials identified as endotoxin positive in the TLR4 reporter assay (LOD: 0.00032 ng/mL). The standard curve for the TLR4 (LPS) reporter assay was fitted using a four-parameter logistic (4PL) regression model: y = 2.16/(1 + (x/0.1427)^−0.81^) with R^2^ = 0.9992. The limit of detection (LOD) is indicated by a black dashed line. Open circles indicate predicted endotoxin concentrations. Nanomaterials listed in the box were classified as TLR4 positive.

For the TLR4 reporter assay, ten materials had activation levels above the detection limit (LOD: 0.00032 ng/mL), indicating the presence of endotoxin contamination. The highest contamination was detected in Bentonite (0.0026 ng/mL), DQ12 (0.0022 ng/mL), NanofilSE3000 (0.0012 ng/mL), NRCWE-20 (0.0012 ng/mL), and NM110 (0.001 ng/mL), while the rest of the tested materials had endotoxin values below 0.001 ng/mL (Figure 3). Notably, all the detected contaminations were below the LOD for the rFC assay (0.005 ng/mL).

### 3.4. Correlation Between rFC and TLR4 Measurements

**Figure 4:**
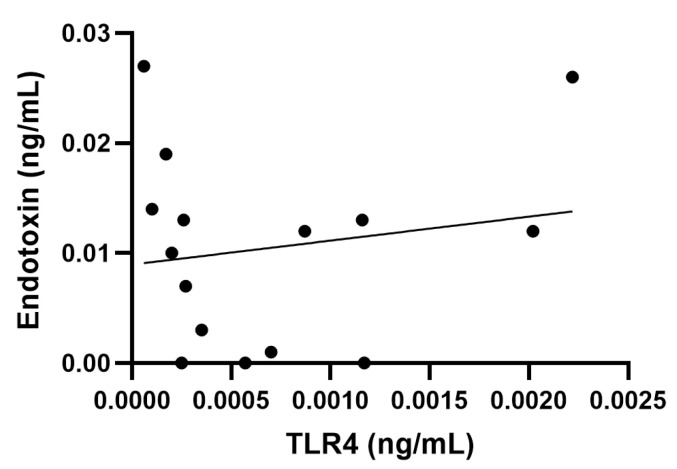
Correlation analysis between endotoxin rFC measurement and toll-like receptor (TLR) 4 activation. No correlation shown (r = 0.17, *p* = 0.5, with a 95% confidence interval −0.38 to 0.63).

Comparison of the measured rFC and TLR4 data showed that, out of the materials with the highest rFC values, DQ12 and rGO were consistently positive for endotoxin across both assays. Moreover, historical LAL data also identified rGO as one of the most contaminated materials (Table 2). The other materials with the highest rFC values showed minimal or no response in the TLR4 assay, i.e., NRCWE-054 (rFC: 0.027 ng/mL; TLR4: <LOD), NRCWE-018 (rFC: 0.019 ng/mL; TLR4: <LOD) and NRCWE-056 (rFC: 0.019 ng/mL; TLR4: <LOD) (Table 2). Aligned with these results, the correlation analysis between endotoxin levels detected by rFC assay and TLR4 cell reporter assay showed an insignificant correlation (r = 0.17, *p* = 0.5, with a 95% confidence interval −0.38 to 0.63) (Figure 4). Finally, all materials that tested positive in the LAL assay, except for Printex90, also had positive endotoxin levels in the rFC assay.

### 3.5. TLR2 Reporter Assay

**Figure 5:**
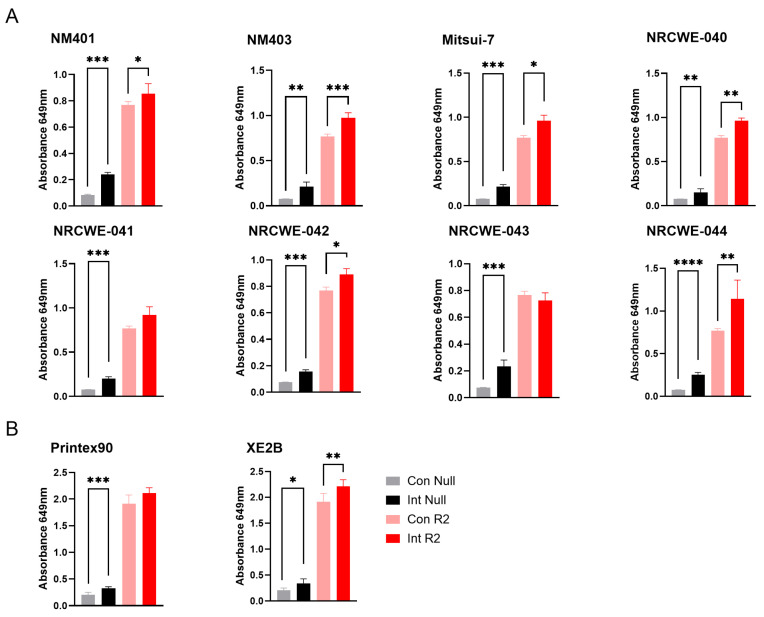
Interference of carbon-based nanomaterials with the TLR4 reporter assay. Interference was assessed at 100 µg/mL nanomaterial concentrations for (**A**) MWCNTs and (**B**) carbon black materials. Con Null and Int Null represent negative control cells without a receptor agonist, in the absence or presence of nanomaterials, respectively. Con R2 and Int R2 represent TLR2 reporter cells stimulated with agonist, without or with nanomaterials, respectively. Data are shown as mean ± SD (*n* = 3). Only statistically significant changes that also exceeded a 20% difference relative to the respective control are shown in the figure. Statistical significance versus corresponding controls is indicated (* *p* < 0.05, ** *p* < 0.01, *** *p* < 0.001, **** *p* < 0.0001). Con = Control, Int = Interference.

Assessment of the interference of nanomaterials with the TLR2 reporter assay showed similar results to the TLR4 reporter assay. Significant interference was observed for all the included MWCNTs as well as the carbon black materials in the absence of TLR2 agonist. Most materials also enhanced the response in the presence of the agonist, except for NRCWE-041, NRCWE-43, and Printex 90 (Figure 5). This indicates that these materials interfere at the wavelength used in the detection of the SEAP signal, suggesting that signal detection methods utilizing other wavelengths may be more suitable for assessment of contaminations on MWCNTs. No or limited interference was observed for the other nanomaterials (Figure S2).

**Figure 6:**
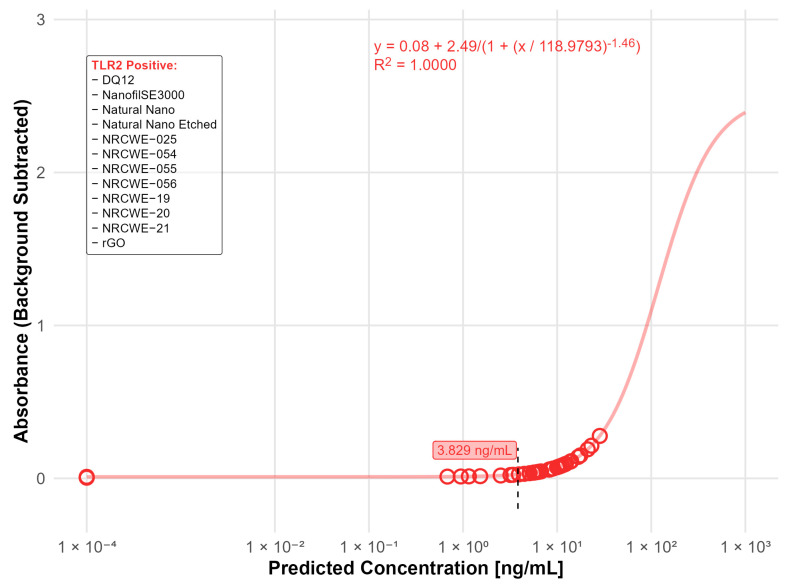
Nanomaterials identified as positive in the TLR2 reporter assay (LOD: 3.83 ng/mL). The standard curve for the TLR2 (LTA) reporter assay was fitted using a four-parameter logistic (4PL) regression model: y = 0.08 + 2.49/(1 + (x/118.9793)^−1.46^) with R^2^ = 1.0000. The limit of detection (LOD) is indicated by the black dashed line. Open circles indicate predicted endotoxin concentrations. The positive nanomaterials were listed in the box.

Of the 20 nanomaterials where no interference was detected, 14 materials were found positive in the TLR2 reporter assay with values above the LOD (3.83 ng/mL). Of these, the highest contaminations were found in DQ12 (24.2 ng/mL) and NanofilSE3000 (12.1 ng/mL) (Figure 6).

## 4. Discussion

Nanomaterials may be contaminated by several microbial components, modifying their immunological effects. Contaminations of nanomaterials with Gram-negative endotoxins are especially concerning due to their ubiquitous presence and resistance to sterilization, leading to misleading results if contamination is not considered [11]. Exotoxins of Gram-positive bacteria are more short-lived and more easily removed by sterilization procedures. While they generally are less potent inducers of macrophage responses, they can trigger neutrophil influx and recruitment [39], making the evaluation of their possible contribution to nanomaterial-induced inflammatory responses important, especially in in vivo studies. Meanwhile, the prevalence of other microbial contaminations, e.g., fungal or viral contaminations on nanomaterials are generally less considered and sparsely reported.

The LAL assay remains the most used method for endotoxin testing of nanomaterials, but the rFC assay is emerging as a promising, sustainable substitute. To date, few studies have utilized rFC for endotoxin testing of nanomaterials, and while the method is accepted as a standardized method for endotoxin testing in the US and EU [40,41], nanomaterial-specific considerations, such as assay interference, still need thorough evaluation. It is well recognized that nanomaterials can distort optical assays through absorption and scattering, thereby biasing readouts [21,42]. To address this, IECs should, according to current international standards, be incorporated into endotoxin tests. In the present study, the commercial rFC assay incorporated IECs to determine material-introduced inhibition or enhancement of the assay signaling. We further implemented an enzyme-free spike-in control to assess matrix-introduced absorbance. Together, these assessments showed that for the tested nanomaterials in this study, no interference was observed, suggesting that the rFC assay may give trustworthy and robust readings on endotoxin contamination for a wide range of different nanomaterial classes. Our data demonstrated that while the assessed nanomaterials generally had low endotoxin values, more than half of the tested nanomaterials had endotoxin levels above the detection limit of the assay, i.e., 0.005 ng/mL corresponding to 0.05 EU/mL. The carbon-based nanomaterials (Printex 90, rGO, NM-401, NM-403, NRCWE-040, NRCWE-041, NRCWE-042, NRCWE-043, NRCWE-044, and NRCWE-045) used in this study were previously tested for endotoxin using the LAL assay [24,38]. While direct comparison is not feasible, these historical data are consistent in showing relatively low endotoxin levels. It is important to note that the assays are different in the chemistry and detection method, and previous comparisons of the two methods have indicated that the rFC gives recovery rates closer to 100%, indicating that rFC may be more suitable than LAL for routine endotoxin testing [43]. It is also important to note that rFC, as well as LAL, does not detect all LPS subtypes equally. Thus, although the rFC assay shows comparable sensitivity to LAL across smooth and rough LPS chemotypes [44], nanomaterial-dependent differential adsorption of LPS subtypes can still bias endotoxin quantification [10] and potentially result in underestimation of the true endotoxin content. Altogether, it should be noted that while the measured endotoxin levels are low, they may still affect sensitive immunological endpoints, particularly when working in vitro with immune cells that are highly responsive to such stimuli. This concern is reinforced by previous studies reporting a high prevalence of endotoxin contamination on nanomaterials used in various EU-funded projects [11], highlighting the need to consider extrinsic factors in the evaluation of nanomaterial effects.

In addition to the regulatory accepted methods for endotoxin testing, several alternative approaches have been suggested. In this work, we focused on the TLR4 reporter assay, which has been shown to perform with similar or higher sensitivity to the chromogenic LAL assay (0.005 EU/mL) [43]. The TLR4 reporter assay has been previously used to assess endotoxin contamination on a small set of nanomaterials, namely TiO_2_, Ag, CaCO_3_, and SiO_2_ [45]. The results suggest a strong potential of the TLR4 reporter assay in the detection of endotoxin and warrant a wider evaluation of its suitability for testing nanomaterial contaminations. In our study, we confirmed that the TLR4 reporter assay can be a good alternative method for endotoxin testing of several types of nanomaterials. However, nanomaterial classes such as carbon-based materials, and especially for the MWCNTs, strong interference was observed at the detection wavelength. This interference likely resulted from the strong and broad light absorbance spectra and high light scatter ability of MWCNTs. In contrast, SWCNTs, which have a narrower absorbance spectrum, showed minimal or no interference. This data suggests that spectral screening of nanomaterials should be included when selecting suitable detection methods to avoid assay artifacts, in addition to maximally removing particles via centrifugation or filtration. TLR reporter assays are offered by several commercial suppliers, utilizing different detection substrates and wavelengths, and some may provide better options for testing materials with high optical activity, such as MWCNTs.

To further investigate these interference patterns, material characterization via DLS was performed, revealing that most nanomaterials maintained stable hydrodynamic sizes across 0 and 24 h in serum-containing culture media, with minimal changes in dispersion behavior. For most metal oxides, ferrites, silica, ZnO, and bentonite, Z-average values in medium and after 24 h differed only modestly, and PDIs were generally centered around 0.4, indicating only moderately aggregating suspensions. In contrast, several high-aspect-ratio CNTs (including Mitsui-7, NM401, NM403, NRCWE-040, -043, -044, and -045) showed dynamic behavior, with lower 24 h Z-averages than initial media values and decreasing PDI (e.g., from 0.59 to 0.42 for NRCWE-043), indicating either partial re-dispersion or, more plausibly for CNTs, agglomeration and sedimentation of large agglomerates outside the DLS detection limit. This dynamic dispersion in MWCNTs correlated with elevated optical interference observed in the TLR4 and TLR2 reporter assays (Figure 2 and Figure 5), where materials like NM403, Mitsui-7, and NRCWE-044 exhibited high absorbance artifacts at 649 nm, potentially due to increased surface exposure, which enhances light absorbance (and associated scattering). DLS data and the interference results together support that the endotoxin values reflect true microbial contamination rather than artifacts from dispersion behavior.

In our study, the TLR4 assay showed high sensitivity to endotoxins with a detection limit of 0.00032 ng/mL, corresponding to 0.003 EU/mL. Our data demonstrates that many of the tested nanomaterials activated the TLR4 reporter assay, indicating the presence of endotoxin contamination. However, the levels were very low, comparable to endotoxin levels below the LODs for rFC and LAL. Interestingly, three of the tested materials, i.e., NRCWE-025, rGO, and Natural Nano Etched, showed activation of the TLR4 reporter, only at the lower nanomaterial concentration. The reason for this is unclear. Notably, this study was designed to use only sub-toxic concentrations of nanomaterials [3,27,36] and the effect was observed exclusively in the TLR4 reporter cells. Nevertheless, caution is warranted in interpreting the results, since the detected endotoxin levels were near the assay’s limit of detection.

While both rFC and TLR4 reporter assays detect endotoxin levels, there are substantial differences in the principles. Important to keep in mind that rFC and also LAL assays measure only the dispersed endotoxin [6], while TLR4 reporter cells may also detect particle-bound endotoxin. Beyond this distinction, the assays also differ fundamentally in how endotoxin is sensed. The rFC assay is an enzymatic reaction primarily responsive to soluble endotoxin, while the TLR4 assay depends on receptor engagement and downstream NF-κB signaling. These pathways vary in their sensitivity to pH, ionic strength, and protein binding, and low-level nanomaterial effects on cell viability or NF-κB activity may further dampen SEAP release. Together, these factors help explain why materials with similar rFC values often showed divergent TLR4 responses, and why the overall correlation between the two assays was weak. Differences in detection limits may also affect the direct comparison of results. Furthermore, it has been suggested that the acute inflammatory effects of graphene oxide may be TLR4-dependent; however, it remains unclear whether this reflects direct receptor binding or secondary activation through alarmin release [19]. Importantly, this TLR4 reporter assay is based on a direct binding and activation of the TLR4, meaning that only biologically available endotoxin would lead to a positive reading in this assay. Thus, TLR4 assays may be a sensitive and useful supplement to classical endotoxin testing; however, thorough interference testing should be integrated as part of contamination assessment strategies, as interference can be affected by many factors such as (i) concentration, (ii) dispersion medium, (iii) wavelength, (iv) particle size and shape, (v) surface chemistry and charge, and (vi) aggregation and stability.

As previously highlighted, microbial contaminations other than endotoxin should also be considered in the interpretation of nanomaterial-induced effects. Microscopy-based techniques such as fluorescence-based bacterial staining and SEM/TEM imaging with immunolabeling can be used to assess the presence of intact bacteria or biofilm; however, these methods have limited sensitivity for detecting low-level or non-viable microbial fragments. MAT does not distinguish between extrinsic microbial-induced effects and intrinsic nanomaterial-induced effects and may also not be suitable for this purpose. On the contrary, TLR2 reporter assays, similar to the TLR4 reporter assay, may provide additional information, as TLR2 recognizes a wide spectrum of microbial Pathogen-Associated Molecular Patterns and plays a central role in sensing Gram-positive bacteria and certain fungal components [46]. However, to comprehensively detect microbial contaminations, including viral or mycoplasma contamination, additional assays would be necessary and provide valuable extensions for future work.

As the TLR2 reporter assay used the same detection substrate and wavelength as the TLR4 reporter assay, interference with the MWCNTs and carbon black materials was also observed here, while the rest of the tested materials did not interfere or had limited interference with the readout. Interestingly, most of the tested materials showed TLR2 activation values above the detection threshold of (3.83 ng/mL). While endotoxins may prime and activate macrophages, and modulate inflammatory responses at very low concentrations [47,48], typical endotoxin acceptance limits for in vitro toxicological and immunotoxicity studies are generally set conservatively below 0.1 EU/mL to avoid false-positive immune activation. To date, there is no suggested threshold for TLR2 activation by microbial ligands. It has been shown that low doses of LTA (5 ng/mL) may prime macrophages and increase their response to subsequent exposures [47]. Furthermore, several TLR2 agonists, such as mycoplasma lipoproteins and synthetic bacterial triacylated lipoproteins, have been shown to trigger macrophage activation (e.g., *Tnf* expression) at 100 ng/mL, and by LTA at 1 µg/mL, whereas neutrophil influx in mice was triggered by TLR2 agonists at 5 or 25 ng/g [49]. Moreover, the synthetic triacylated lipoproteins Pam2CSK4 can trigger TNF secretion from macrophages already at concentrations of 0.1 ng/mL [50]. Thus, it is difficult to predict the biological effects of TLR2 activation as different agonists have different potencies and response patterns.

Notably, TLR2 signaling may occur both from intrinsic nanomaterial-driven and from contaminant-derived activations. While acknowledged as a limitation, it is unlikely that the materials tested in this study directly activate the TLR2 assay, as previous in vivo data showed largely TLR2-independent responses for the tested carbon black, CNTs, and graphene oxide materials in inducing pulmonary inflammation in TLR2 knockout mice [19]. This supports the interpretation that the TLR2 activity detected in vitro is more plausibly explained by the presence of microbial ligands rather than by strong intrinsic TLR2 activation by the nanomaterials themselves. However, to ensure correct interpretation, parallel testing (rFC + LAL), spiking/recovery controls with both LPS chemotypes and relevant TLR2 ligands, and mechanistic follow-up studies are recommended, especially for suspected TLR2-activating nanomaterials to ensure accurate attribution of observed immune effects. Altogether, this highlights both the potential and the limitations of the TLR2 reporter-based approaches for contamination testing. When applied as a complementary tool for detecting contamination, with the inclusion of appropriate controls, it can provide useful additional information, though it should not be relied upon as the sole indicator of nanomaterial microbial contamination.

Finally, in the assessment of nanomaterial contamination, it is important to remember that co-exposure to microbial and particulate materials can produce biological responses that are not simply additive. Adsorption of LPS to particle surfaces and particle-driven changes in uptake or signaling can shift cytokine profiles and activation states across neutrophils, macrophages, and dendritic cells, with both enhancement and suppression observed depending on particle chemical property and dose [51,52,53,54]. Prior particulate exposure can also reprogram subsequent responses to endotoxin, consistent with immune priming or tolerance [55,56]. Similarly, Gram-positive bacterial components can interact with particles and modulate immune response. Co-exposure to ultrafine carbon black particles and LTA has been shown to potentiate pulmonary inflammation and enhance neutrophilic responses [57]. Co-exposure to zinc- and copper-containing particles and LTA enhances macrophage inflammatory responses via TLR2 signaling [58]. These findings demonstrate that effects cannot be attributed solely to the particle with confidence unless microbial contaminants are excluded; validated assays should therefore be applied and reported to ensure that outcomes reflect material properties rather than undetected microbial contaminants.

Altogether, we suggest that future nanotoxicological evaluations should consistently include a comprehensive assessment of microbial contaminations, preferably utilize multiple testing methods, and incorporate suitable interference controls. We propose a practical workflow in which rFC serves as the primary biochemical assay for assessment of soluble endotoxins, followed by TLR4 and TLR2 reporters to capture additional biologically active microbial contaminants. For carbon black and multi-walled carbon nanotubes, special care should be taken to eliminate optical assay interference. Adopting this multi-assay strategy will improve the robustness of microbial contamination assessment in nanotoxicology to ensure accurate interpretation of immunological responses.

## Figures and Tables

**Table 1:** Included nanomaterials, their characteristics, and hydrodynamic diameters.

Material	ID	Size (nm) ^1^	Aspect Ratio	BET	DLS (Stock)		DLS (Media)		DLS (Media 24 h)	
					Z-ave	PDI	Z-ave	PDI	Z-ave	PDI
Carbon black	Printex90	14		338	106.3	0.24	129.1	0.20	120.3	0.17
Carbon black	XE2B	30		1142	179.7	0.22	237.8	0.23	217.5	0.18
Reduced graphene oxide ^2^	rGO	1.7 × 1500		411	547.5	0.45	351.4	0.54	355.4	0.52
MWCNT	Mitsui-7	67 × 5600	83.6	26	672.6	0.34	1095.5	0.41	743.7	0.37
MWCNT	NM401	67 × 4048	60.4	18	697.9	0.33	1303.5	0.31	1161.1	0.40
MWCNT	NM403	12 × 443	36.9	135	198.6	0.42	576.3	0.52	388.5	0.48
MWCNT	NRCWE-040	22 × 519	23.6	150	180.6	0.39	541.5	0.49	275.3	0.42
MWCNT	NRCWE-041	27 × 1005	37.2	152	172.3	0.36	284.8	0.46	277.1	0.45
MWCNT	NRCWE-042	30 × 723	24.1	141	190.3	0.42	203.9	0.35	199.6	0.40
MWCNT	NRCWE-043	56 × 771	13.8	82	175.5	0.30	695.8	0.59	308.4	0.42
MWCNT	NRCWE-044	33 × 1330	40.3	74	195.6	0.29	487	0.50	314.0	0.39
MWCNT	NRCWE-045	30 × 1553	51.8	119	197.9	0.27	358.2	0.36	312.8	0.38
SWCNT	NRCWE-054	1 × 17,500	17,500	370.8	1025	0.87	374.9	0.76	270.0	0.53
SWCNT	NRCWE-055	1 × 2000	2000	453.1	606.2	0.89	452.8	0.84	550.1	0.82
SWCNT	NRCWE-056	1 × 2000	2000	373.4	502.5	0.87	337	0.77	458.8	0.86
TiO_2_ anatase	MKN-A015	31		84.6	177.1	0.38	221.6	0.39	211.2	0.40
TiO_2_ anatase	MKN-A100	22		73.5	149.8	0.40	174.2	0.40	179.4	0.38
TiO_2_ rutile	NRCWE-001	10		99	115.1	0.18	131.3	0.17	132.5	0.17
TiO_2_ rutile	NRCWE-025	38		28.2	190.5	0.17	213.9	0.13	222.4	0.16
TiO_2_ rutile	NRCWE-030	11		139.1	116.4	0.19	133.4	0.16	133.4	0.17
Fe_2_O_3_	NRCWE-018	40		27.7	136.4	0.16	151.2	0.17	154.2	0.17
Fe_2_O_3_	NRCWE-019	85 × 425	5.0	27.4	253.9	0.17	322.4	0.18	317.5	0.17
NiZnFe_4_O_8_	NRCWE-020	20		104	341.9	0.45	248.9	0.44	248.5	0.45
ZnFe_2_O_4_	NRCWE-021	23		87.7	406.2	0.37	403.0	0.48	611.9	0.46
NiFe_2_O_4_	NRCWE-022	25		86.9	245.6	0.37	246.8	0.39	238.6	0.36
ZnO	NM110	85		14	214	0.13	210.4	0.22	189.3	0.26
SiO_2_	DQ12	225		10.1	430.9	0.28	351.9	0.54	383.8	0.81
Nanoclay ^2^	Bentonite	7.8 × 650		73.6	284.3	0.45	158.6	0.43	163.1	0.42
Nanoclay ^2^	NanofilSE3000	1 × 300		2.7	1077	0.68	102.2	0.40	351.7	0.49
Halloysite nanotube	Natural Nano	61 × 250	4.1	25.6	266.3	0.16	277.6	0.28	244.0	0.65
Halloysite nanotube	Natural Nano Etched	27 × 100	3.7	128	497.2	0.41	219.3	0.71	283.9	0.29

^1^ Size information obtained from [2,23,24,25,26,27,28,29,30,31,32,33,34,35]. ^2^ Exhibits plate-like morphology. Abbreviations: MWCNT, multi-walled carbon nanotube; SWCNT, single-walled carbon nanotube; DLS, dynamic light scattering; PDI, polydispersity index.

**Table 2:** Summary of endotoxin levels measured by rFC, LAL, and TLR reporter assays.

Material	ID	rFC (ng/mL)	LAL ^1^ (ng/mL)	TLR4 (ng/mL)
Carbon black	Printex90	0.000	0.165	na ^2^
Carbon black	XE2B	0.006	na	na
Reduced graphene oxide	rGO	0.028	2.680	0.009
MWCNT	Mitsui-7	0.002	na	na
MWCNT	NM401	0.001	1.350	na
MWCNT	NM403	0.012	0.035	na
MWCNT	NRCWE-040	0.001	0.460	na
MWCNT	NRCWE-041	0.012	2.042	na
MWCNT	NRCWE-042	0.009	0.670	na
MWCNT	NRCWE-043	0.008	0.650	na
MWCNT	NRCWE-044	0.017	0.690	na
MWCNT	NRCWE-045	0.012	0.870	0.057
SWCNT	NRCWE-054	0.027	na	<LOD ^3^
SWCNT	NRCWE-055	0.003	na	<LOD
SWCNT	NRCWE-056	0.019	na	<LOD
TiO_2_ anatase	MKN-A015	0.007	na	0.006
TiO_2_ anatase	MKN-A100	0.014	na	<LOD
TiO_2_ rutile	NRCWE-001	0.001	na	0.021
TiO_2_ rutile	NRCWE-025	0.010	na	0.008
TiO_2_ rutile	NRCWE-030	0.001	na	0.026
Fe_2_O_3_	NRCWE-018	0.019	na	<LOD
Fe_2_O_3_	NRCWE-019	0.015	na	<LOD
NiZnFe_4_O_8_	NRCWE-020	0.013	na	0.041
ZnFe_2_O_4_	NRCWE-021	0.004	na	<LOD
NiFe_2_O_4_	NRCWE-022	0.000	na	<LOD
ZnO	NM110	0.000	na	<LOD
SiO_2_	DQ12	0.026	na	0.050
Nanoclay	Bentonite	0.001	na	0.010
Nanoclay	NanofilSE3000	<LOD	na	0.033
Halloysite nanotube	Natural Nano	0.012	na	0.032
Halloysite nanotube	Natural Nano Etched	0.000	na	<LOD

^1^ Endotoxin level detected by LAL assay obtained from Bengtson S. et al., 2016 [24], and Jackson P. et al., 2015 [38]; ^2^ na: TLR4 assay not performed because these materials have strong optical interference in the reporter assay; ^3^ < LOD: Below the limit of detection of the TLR4 assay.

## Data Availability

The original contributions presented in this study are included in the article/Supplementary Material. Further inquiries can be directed to the corresponding authors.

## Supplementary Materials

The following supporting information can be downloaded at: https://www.mdpi.com/article/10.3390/nano15241871/s1, Table S1. Interference with the rFC assay; Figure S1. MWCNT interference at different dilutions in receptor cell assays; Figure S2. Interference with cell reporter assays for SWCNT, metal-based, silica, and graphene materials.

## Abbreviations

The following abbreviations are used in this manuscript:

ANOVA	Analysis of variance
BSA	Bovine serum albumin
DLS	Dynamic light scattering
HNT	Halloysite nanotubes
HPLC-MS	High performance liquid chromatography and mass spectrometry
IECs	Inhibition-enhancement controls
LAL	Limulus amoebocyte lysate
LOD	Limit of detection
LTA	Lipoteichoic acid
MAT	Monocyte activation test
MWCNT	Multi-walled carbon nanotube
PDI	Polydispersity index
rFC	Recombinant Factor C
SEAP	Secreted alkaline phosphatase
SEM	Scanning electron microscopy
SWCNT	Single-walled carbon nanotube
TEM	Transmission electron microscopy
TLR	Toll-like receptor
