# Supplementary material for: Detection of Microbial Contamination in Nanomaterials Using LAL, rFC and Cell-Based Assays: Implications for Nanotoxicological Hazard Assessment
Source: Nanomaterials (Basel). 2025 Dec 13;15(24):1871. doi: 10.3390/nano15241871 (PMC12736212; doi:10.3390/nano15241871)
Supplement: Supplementary file 1 [file nanomaterials-15-01871-s001.zip › Supplementary Figure captions.pdf]

Figure S1. Interference with rFC assay; Figure S1: MWCNT interference at different dilutions in receptor cell assays. Interference was evaluated in HEK-Blue™ TLR2 (R2) and TLR4 (R4) cells following the addition of multi-walled carbon nanotubes at 10, 50, and 100 µg/mL. Bars show absorbance for control wells without agonist (Con Null), interference controls (Int Null), and TLR2/4 agonist-stimulated wells with (Int) or without (Con) nanoparticles (Con R2, Int R2, Con R4, and Int R4). Data = mean ± SD (n ≥ 3). *p < 0.05; **p < 0.01; ***p < 0.001, ****p < 0.0001.

Figure S2. Interference of SWCNTs, metal-based nanoparticles, nanoclays, graphene, and silica particles with cell reporter assays.
Optical interference during SEAP detection (649 nm) was assessed in HEK-Blue™ TLR2 (R2) and TLR4 (R4) cells. Bars show absorbance for control wells without agonist (Con Null), interference controls (Int Null), and TLR2/4 agonist-stimulated wells with (Int) or without (Con) nanoparticles (Con R2, Int R2, Con R4, and Int R4). (A) SWCNTs (NRCWE-051, -054, -055, and -056); (B) metal-based NPs (NRCWE-18, -19, -20, -21, -22, NM-110, NRCWE-030, -001, -025, MKN-A015, and MKN-A100); (C) nanoclays (Bentonite, Nanofil 3000, Natural Nano, Natural Nano Etched); (D) graphene (rGO); (E) silica (DQ12). Data = mean ± SD (n ≥ 3). *p < 0.05; **p < 0.01.
