# Supplementary figures and images for: Detection of Microbial Contamination in Nanomaterials Using LAL, rFC and Cell-Based Assays: Implications for Nanotoxicological Hazard Assessment
Source: Nanomaterials (Basel). 2025 Dec 13;15(24):1871. doi: 10.3390/nano15241871 (PMC12736212; doi:10.3390/nano15241871)

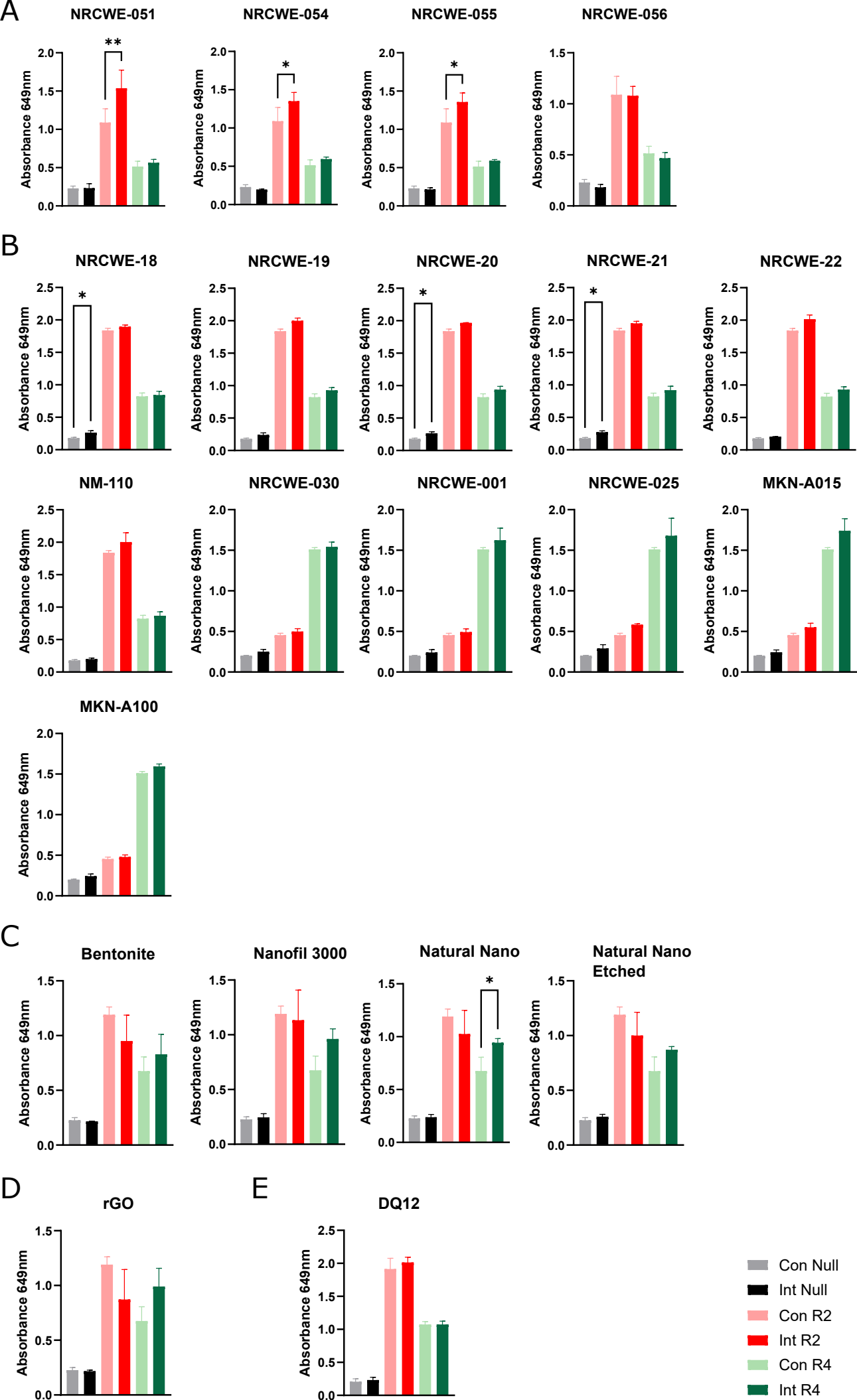

Supplement: Supplementary file 1 [file nanomaterials-15-01871-s001.zip › Figure S1.pdf]

NM401

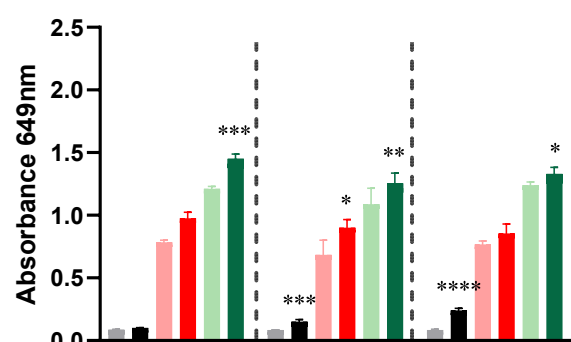

NM403

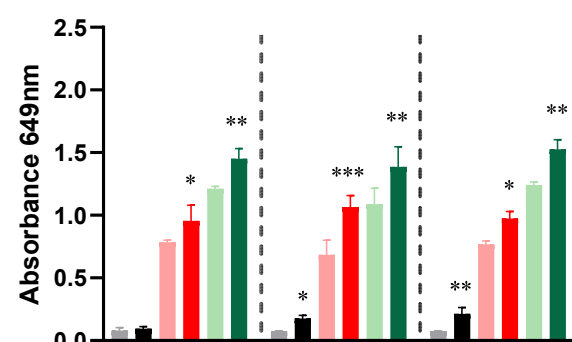

Mitsui-7

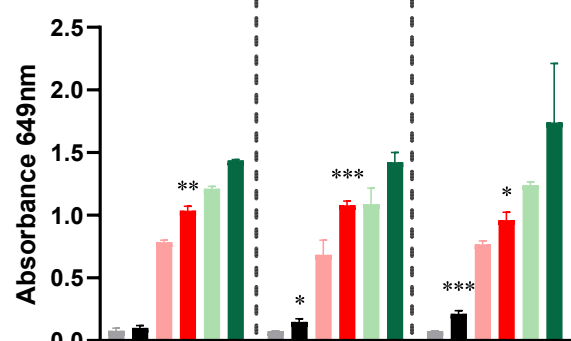

NRCWE-40

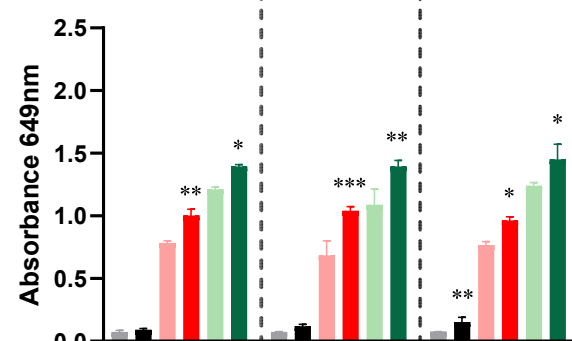

NRCWE-041

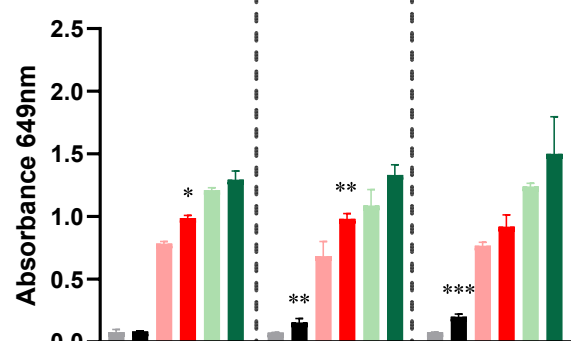

NRCWE-042

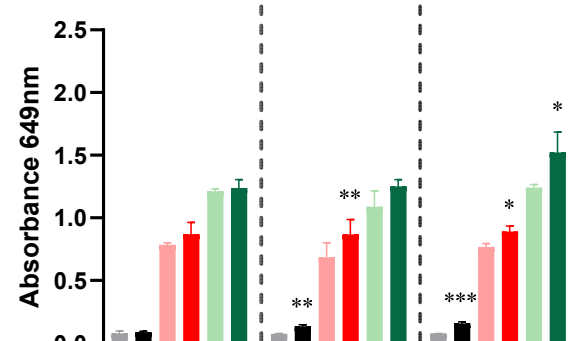

NRCWE-043

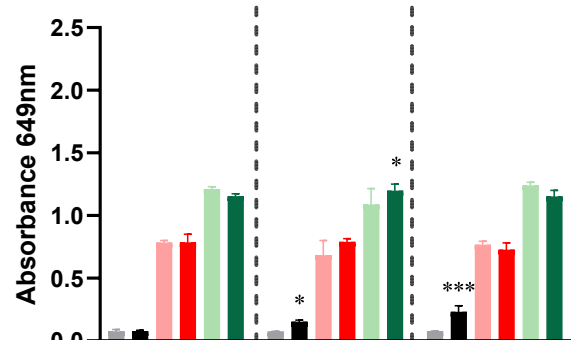

NRCWE-044

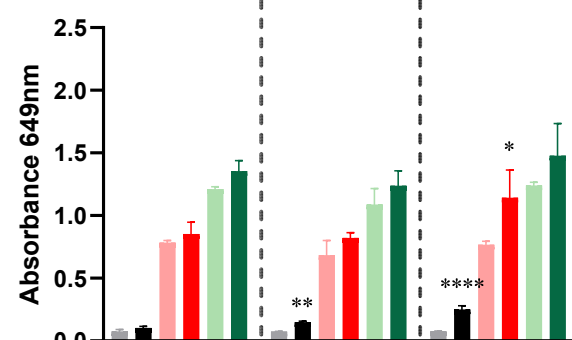

Con Null  
Int Null

Con R2  
Int R2

Con R4  
Int R4

Supplement: Supplementary file 1 [file nanomaterials-15-01871-s001.zip › Figure S2.pdf]
